# Stability, Permeability and Cytotoxicity of Buccal Films in Allergy Treatment

**DOI:** 10.3390/pharmaceutics14081633

**Published:** 2022-08-05

**Authors:** Krisztián Pamlényi, Géza Regdon, Dániel Nemes, Ferenc Fenyvesi, Ildikó Bácskay, Katalin Kristó

**Affiliations:** 1 Institute of Pharmaceutical Technology and Regulatory Affairs, University of Szeged, H-6720 Szeged, Hungary; 2 Department of Pharmaceutical Technology, University of Debrecen, H-4032 Debrecen, Hungary

**Keywords:** mucoadhesive, buccal films, drug delivery, alginate, cetirizine, antihistamine, stability, permeability, cytotoxicity

## Abstract

Oral mucoadhesive systems, such as polymer films, are among innovative pharmaceutical products. These systems can be applied in swallowing problems and can also be used in geriatrics and paediatrics. In our earlier work, we successfully formulated buccal mucoadhesive polymer films, which contained cetirizine-hydrochloride (CTZ) as the API. The present study focused on investigating the stability and permeability of the prepared films. The stability of the films was studied with an accelerated stability test. During the stability test, thickness, breaking hardness and in vitro mucoadhesivity were analysed. Furthermore, the interactions were studied with FT-IR spectroscopy, and the changes in the amount of the API were also monitored. Cytotoxicity and cell line permeability studies were carried out on TR 146 buccal cells. Compositions that can preserve more than 85% of the API after 6 months were found. Most of the compositions had a high cell viability of more than 50%. Citric acid (CA) decreased the stability and reduced every physical parameter of the films. However, cell line studies showed that the permeability of the films was enhanced. In our work, we successfully formulated CTZ-containing buccal films with adequate stability, high cell viability and appropriate absorption properties.

## 1. Introduction

An allergy is a condition caused by the hypersensitivity of the immune system, an increased sensitivity that results from a harmful substance triggering an immunological response [1]; it is also known as allergic disease. Allergies affect more and more people all around the world [2]. The symptoms of allergic diseases may include red skin, red eyes, a runny nose, sneezing, shortness of breath or swallowing problems. Medications can be used to improve allergy symptoms [1,3,4].

The most commonly applied medications in the treatment of allergies are antihistamines, which can block the H1 receptor [4]. Cetirizine dihydrochloride (CTZ) is a second-generation, non-sedative antihistamine [5,6]; it is the active metabolite of the first-generation H1 receptor blocker, hydroxyzine [7]. Due to its antihistamine properties, it can be used effectively in the treatment of seasonal or chronic allergic rhinitis and urticaria [7,8]. Some studies reported that it can reduce bronchoconstriction and swallowing problems. CTZ is available on the market in tablets and solution dosage forms [9]. The patient must always swallow the dosage form, but it is impossible if there is a swallowing problem due to laryngeal oedema. In this case, the patient has to apply an injection or wait for medical help.

Buccal polymer films offer an alternative and innovative way to deliver an active pharmaceutical ingredient (API) to the systemic circulation without swallowing the dosage form [10]. The application of buccal films is easy and not painful, and patients do not feel a foreign body sensation. Therefore, in geriatrics and paediatrics, they could offer great advantages over per os tablets and suppositories [11,12]. At the same time, a lower dose of the API can be used compared with the listed dosage forms. The first-pass effect of the liver is avoided, and the API can enter the systemic circulation directly from the buccal mucosa. The API and gastric acid do not contact each other in the case of buccal drug administration, so the stomach can be protected from the API and vice versa [13,14,15]. Buccal films as a dosage form are not official in the Pharmacopoeias [16]. However, the pharmaceutical industry has already started to focus on buccal films and recognized them as a potential drug delivery system [17], as evidenced by the availability on the market of a buccal film called Breakyl^®^, containing fentanyl [18]. Many research groups have tried to formulate buccal polymers films with different APIs, such as omeprazole, miconazole, ondansetron and prednisolone [19,20,21,22]. Transcellular and paracellular drug delivery across the lipid membrane of the buccal mucosa is equally good, which can result in good transport for hydrophilic and lipophilic APIs [23]. The bioavailability of the API can be enhanced with films; the API bypasses the liver, so the first-pass effect of the liver does not influence the pre-metabolization of the API [24]. At the same time, the API can be protected from the degradation of the gastrointestinal tract [25]. Therefore, the entire amount of the permeated API can have an effect. In our case, CTZ can act immediately after permeation, so it can quickly reduce serious symptoms without medical help. Further advantages of buccal films are that they are painless and easy to apply; patients can use them easily compared with an injection [26]. Summarizing these facts, buccal films containing CTZ are able to reduce and moderate allergic symptoms in a short time, and depending on the formulation, they are also capable of protecting allergic patients from a life-threatening condition.

In our earlier work, we formulated buccal films with sodium alginate (SA), HPMC and glycerol (GLY), partly with the composition that appears in this research [15]. In that work, the physical properties of the prepared films were studied, such as thickness, tensile strength and in vitro mucoadhesion. Statistical analysis was applied to predict the optimal composition. In addition, the chemical interactions were studied, and the homogeneity of the API was also investigated in the different compositions. Finally, the API content was measured [15]. Most of the compositions were found to be promising, so we found them worthwhile to investigate further.

Sodium alginate (SA) is a linear polysaccharide from a natural source, extracted from brown seeds. SA has hydroxyl and carboxyl groups, which can bind to the mucin of the buccal mucosa, so it is a mucoadhesive polymer [19]. In pharmaceutical technology, it can be used as a polymer film-forming agent to develop buccal films [15,19]. In addition, it is used as a coating agent as well [27]. HPMC is an old conventional material in the pharmaceutical industry. It is a water-soluble polymer. It has a larger number of functional groups (methoxy, carboxyl, hydroxyl), so it can form stable H-bonds with the mucin [28,29]. It is applied as a film-forming agent and a coating agent, and it is often the base of the matrix system in tablets, gels and eye drops [30]. Glycerol (GLY) is a well-known, colourless, viscous, cytocompatible liquid. GLY is widely used in various fields. In pharmaceutical technology and polymer chemistry, it is applied as a plasticizer because it can increase the flexibility and elasticity of film and the coating [31].

The aim of our current research is to investigate the physical and physicochemical stability of the prepared films, which had a promising composition based on our earlier research work [15]. The investigation of the stability of oral films is a less explored area of buccal film research, so it can be stated that it is definitely the novelty of our article. During this period, many processes may take place in the film, and we aimed to examine them. Based on data from the literature, citric acid (CA) enhances permeation [32,33]; therefore, it was incorporated into the films to enhance permeation. Moreover, we also examined the permeation of CTZ from buccal films across an artificial membrane and the TR 146 buccal cell line as well, which are also novelties in our work. The dissolution test is extremely important, but the knowledge of cell permeation is essential for the effect to occur; therefore, we set a goal to investigate it. Cytotoxicity is also an important investigation because it may influence the outcome of the further development of the dosage form. Finally, based on our results, we aimed to select the best composition with good stability, high permeability and appropriate cytotoxicity.

## 2. Materials and Methods

### 2.1. Materials

Sodium alginate (SA) (Biochemica GmbH, Darmstadt, Germany) (10,000–600,000 g/mol) and hydroxy methylcellulose (HPMC) (Pharmacoat^®^ 603, Shin Etsu Chemical Co., Ltd., Tokyo, Japan) were used as film-forming agents in the polymer film. Glycerol (GLY) 85% (*w*/*w* %) was added to the film as a plasticizer (Ph. Eur.8.). Citric acid (CA) (Ph. Eur. 8.) was incorporated in the polymer film system as a permeation enhancer. Cetirizine dihydrochloride (CTZ) (Ph. Eur. 8.) was the API, which was a gift from ExtractumPharma Pharmaceutical Manufacturing, Marketing and Consulting Inc., Kunfehértó, Hungary. Mucin (Carl Roth GmbH + Co. KG, Karlsruhe, Germany) (10 *w*/*w* %) dispersion was used in the in vitro mucoadhesion test.

### 2.2. Preparation of Buccal Films

Samples 5, 7, 9 and 11 (Table 1) were prepared and tested for their physical and physicochemical parameters in our previous work [15]. Based on this study, these samples proved to be promising compositions during the optimization process; therefore, we continued to work with these compositions in our present work. In this work, the CA-free samples and CA-containing films were also reproduced so that the effect of CA could be examined during the comparison. In addition, we studied how the parameters change in the case of purely SA-based films without HPMC (Samples 1–4).

The films were prepared at room temperature with the solvent casting method. As the first step of preparation, SA (1.5, 2, 3 *w*/*w* %) was dissolved in distilled water and mixed (900 rpm) at room temperature. The solution was heated to 70 °C and mixed (900 rpm), and CTZ was then incorporated in the warm solution (70 °C, 0.5523 g/100 g solution), and the mixing was continued for 5 h. As the third step, HPMC (0, 1, 1.5 *w*/*w* %) and CA were added to the solution with mixing without heating. In the fourth step, GLY was added to the solution the following day. Mixing was decreased to 100 rpm for 3 h to help the air bubbles disappear from the solution. The solution was cast on a glass surface in Petri dishes, with 10 g of solution/dish; then, it was dried at room temperature (24.4 ± 0.5 °C). The dried polymer films were removed from the surface and were placed in closed containers (24.4 ± 1 °C, 60 ± 2% RH), and the other part of the prepared films was also placed in closed containers, but at 40 °C ± 2 °C, 75% RH ± 5% RH. The prepared films contained 10 mg of CTZ on an area of 4 cm^2^, which is the therapeutic dose of this API.

Table 1 shows the different compositions of the prepared polymer films. Every film composition contains CTZ, and every second film composition contains CA.

### 2.3. Stability Test

During product development, it is essential and unavoidable to assess the stability of the drug and the drug delivery system. In our research work, stability studies were done according to ICH guidelines. The prepared films were placed in closed containers and stored at 40 °C ± 2 °C, 75% RH ± 5% RH for a period of 6 months. Different methods were applied to obtain information about the changes in the properties of the films. During the stability test, thickness, breaking hardness, in vitro mucoadhesivity and permeability were analysed to determine changes in the physical properties of the films. Furthermore, the interactions were also examined with FT-IR spectroscopy, and the drug content was also detected during the 6-month period.

#### 2.3.1. Breaking Hardness of Films

Breaking hardness was measured with a texture analyser developed in our department [34]. The equipment has different sample holders and two different probes (needle-like probe, rod-like probe). The equipment has two parts, a fixed disc and a moving sample holder. Depending on the measurement, a different sample holder can be applied, and during the investigation, the force, moving speed and time can be registered and followed. The needle-like probe was applied in the breaking hardness test. The probe went to the sample, which was fixed on the bottom part of the equipment. The probe went through the film, and the equipment detected the time and force during the investigation. The test was finished when the film was broken. The test was done six times (*n* = 6) for each combination of films. The means and standard deviations were calculated. The equipment was presented in our earlier research papers [15,33,34].

#### 2.3.2. In Vitro Mucoadhesivity of Films

Mucoadhesion was investigated with the same texture analyser with different settings and parameter modifications [15,35]. In this study, a rod-like sample holder with a diameter of 5 mm was used. A double-faced adhesive tape was fixed on the surface of the sample holder, and the polymer film was fixed to the other face of the adhesive tape. On the bottom part of the tester, a fixed disc with a diameter of 35 mm was applied, and 40 µL of freshly prepared mucin dispersion (10 *w*/*w* %) was spread on it. The rod-like sample holder went to the fixed, bottom disc and pressed the polymer film to the mucin-covered bottom disc with 30 ± 0.1 N for 30 s. This steady-state part can be found in the force–time curve. Thereafter, the sample holder went back to the original place, and the force was decreased until the sample started to separate from the mucin, which can be seen as a well-defined, sharp peak in the force–time curve, indicating the in vitro mucoadhesion force of the films. The test was done six times (*n* = 6), and the means and standard deviations were calculated.

#### 2.3.3. Active Agent Content

In the determination of API content, 4 cm^2^ polymer films (containing 10 mg of CTZ) were studied. The test was carried out by an Erweka DT700 dissolution basket tester at a mixing speed of 100 rpm. An amount of 900 mL of phosphate buffer (pH = 6.8) was used as dissolution medium; its temperature was 37 °C [36]. Six parallel measurements were performed. Aliquots of 5 mL were analysed at 120 min with Genesys 10S UV–VIS (Thermo Fisher Scientific, Waltham, MA, USA) UV-spectrophotometry at λ = 207 nm wavelength.

#### 2.3.4. FT-IR Spectroscopy

An Avatar 330 FT-IR apparatus (Thermo-Scientific, Waltham, MA, USA) was used to analyse the Fourier-Transform Infrared Spectra of the raw materials and the prepared polymer films. The apparatus was equipped with a coupled Zn/Se horizontal attenuated total reflectance (HATR) unit. The films were put on a clean crystal of the apparatus. The range of wavelength was 600 to 4000 cm^−1^ during the investigation. The spectra were collected from 128 scans at the spectral resolution of 4 cm^−1^ with CO_2_ and H_2_O for correction.

### 2.4. Permeation Test

The permeation property of CTZ was investigated with two different permeation methods. As the first step, in the Enhancer Cell, the permeation of the API was examined from the polymer films across an artificial cellulose acetate membrane. In the next step, the permeation of the API was measured on the TR146 buccal cell line under in vitro conditions [37,38,39].

#### 2.4.1. Permeation Test across Artificial Membrane

The permeation of CTZ was studied in the Enhancer Cell across an artificial cellulose acetate membrane (Whatman^®^, SN:WHA10404106, pore size 0.2 µm, surface 2.54 cm^2^) [40]. The size of the measured films was 1 × 1 cm. The films were put on the surface of the membrane and placed into the donor phase, which was 2 mL phosphate buffer (pH = 6.8), modelling saliva. The acceptor phase was also phosphate buffer (pH = 7.4, 300 mL), which simulated the pH of blood. The test was run in an Erweka DT700 dissolution basket tester at a mixing speed of 100 rpm. Aliquots of 5 mL were analysed at 15, 30, 60, 90, 120, 180 and 240 min with Genesys 10S UV–VIS (Thermo Fisher Scientific, Waltham, MA, USA) UV-spectrophotometry at λ = 207 nm wavelength.

#### 2.4.2. Cell Culturing

TR-146 cells (European Collection of Authenticated Cell Cultures Catalogue No: 10032305) were cultured in Dulbecco’s modified Eagle’s medium with l-glutamine and d-glucose supplemented with 10% (*v*/*v*) FBS, 3.7 g/L sodium hydrogen carbonate, 1% (*v*/*v*) non-essential amino acid solution and 100 IU/mL penicillin K, with 100 μg/mL streptomycin sulfate at 37 °C in an atmosphere of 5% CO_2_ in plastic cell culture flasks. The cells were routinely maintained by regular passaging. Cells used for our experiments were between passage numbers 20 and 28.

#### 2.4.3. In Vitro Permeation Test

For the permeation test, 1 × 10^5^ cells were seeded on ThinCert^®^ PET cell culture inserts with a pore size of 0.4 µm, a pore density of 2 × 10^6^/cm^2^ and a culturing surface of 33.6 mm^2^ (Greiner BioOne, Mosonmagyaróvár, Hungary). The culture medium was changed twice per week on the inserts, and the cells were grown for 2 weeks in 24-well plates. A Millicell ERS 1 device was used to measure transepithelial electrical resistance (Merck, Budapest, Hungary). After 2 weeks, only inserts with a resistance value over 130 Ω·cm^2^ were used, which correlates well with previous reports [41]. The films were cut to equal weight with an overall CTZ content of 0.35 mg. Each film was dissolved in Hank’s balanced salt solution (HBSS) with pH = 7.2 before the experiment. The cell culture medium was removed from the inserts, and 400 µL of the dissolved films was added to the apical compartment (AC) and 1000 µL to the basolateral compartment (BC). After 30, 60 and 90 min, 2 × 100 µL of solution was removed from the wells after gentle mixing. The removed volume was replaced with HBSS. The samples were placed into UV-Star 96-well plates (Greiner BioOne, Mosonmagyaróvár, Hungary), and their absorbance was measured at 248 nm with a Multiskan Go microplate reader (Thermo-Fisher, Waltham, MA, USA). Apparent permeability (Papp) was calculated as: (ΔQ/Δt) × (1/(0.336 cm^2^ × C_0_)), where C_0_ is the concentration of the tested compound (mg/mL); ∆Q/∆t is the rate of permeability of the tested compound ((mg/mL)·s^−1^) between 60 and 90 min.

### 2.5. Cytotoxicity Test

In order to study the cytotoxicity of CTZ films, a Guava^®^ easyCyte™ 5HT (Luminex, Austin, TX, USA) flow cytometer was used for our experiments. TR-146 cells were collected from cell culture flasks with a trypsin–EDTA solution and redistributed into separate tubes, and 1 million cells were treated with 1 mL of CTZ solution (CTZ films dissolved in HBSS in equal concentration as in the case of the permeation tests). After 30 min of incubation, the cells were centrifuged, the test solutions were removed, and the cells were gently washed with cold HBSS and centrifuged again. The supernatant was removed, and a 1 million cells/mL cell suspension was prepared with HBSS and then stained with 1 µL of 100 µg/mL propidium iodide solution. After 15 min, the suspensions were distributed on 96-well microplates in a volume of 200 µL (3 wells/group) and analysed. Propidium iodide was excited with a 488 nm laser and detected at the 525/30 nm channel (green parameter). On the Forward and Side Scatter Patterns (FSC-SSC) scatterplot, the non-cellular events were excluded. The remaining events (8000–10,000) were analysed on a scatterplot, and gates were created to determine stained (necrotic) and non-stained (living) cells [42]. The experiment was carried out in triplicate. As negative control, HBSS was used, and cells treated with HBSS instead of the dissolved films were considered 100%, to which all treated groups were compared. As positive control, cells were treated with 1% Triton-X 100.

### 2.6. Statistical Analyses

The significance tests for breaking hardness and in vitro mucoadhesivity were performed with Microsoft Excel (version 15, Redmond, Washington, DC, USA) software. A Two-Sample T-Test was applied. The test was done six times for each sample. In all cases, the samples were compared to the freshly prepared sample (0 months). The apparent permeability values of the films were analysed with the Kruskal–Wallis test with Dunn’s test as post hoc test. In each case, we used a significance level of *p* < 0.05. Significance is labelled as ns = *p* ≥ 0.05; * = *p* < 0.05 ** = *p* < 0.01.

## 3. Results

### 3.1. Stability Test 

#### 3.1.1. Breaking Hardness of Films

**Figure 1:**
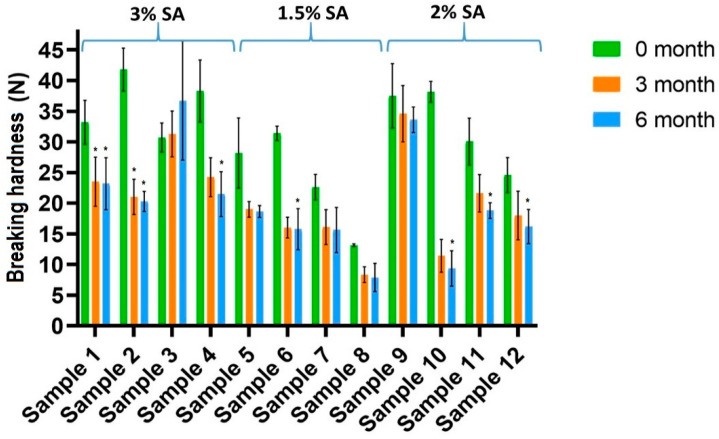
Breaking hardness of the prepared films (Samples 2, 4, 6, 8, 10, 12 contain CA) (* *p* < 0.05; *n* = 6). The samples were compared to the freshly prepared sample (0 months).

The results of the breaking hardness measurements are presented in Figure 1. The significance results are marked with * (*: *p* < 0.05). The changes in the extent of the breaking hardness of the films during the 6-month period are marked in different colours. Breaking hardness decreased for every composition during the period. At the beginning of the investigation, the breaking hardness of the films containing CA was higher than that of the films without CA. This observation could be explained by the fact that CA can form cross-linking and hydrogen bonds with the other components of the films; therefore, CA-containing films have a stronger, cohesive structure [43]. On the other hand, Uranga et al. found that CA can have a plasticizer effect in the polymer film, which can confirm the higher force in the freshly prepared sample; therefore, these films are flexible [44]. At the same time, in the case of CA-containing films (Samples 2, 4, 6, 8, 10, 12), the decrease in breaking hardness was higher than without CA (Samples 1, 3, 5, 7, 9, 11). As the GLY concentration increased, the tensile strength of the films decreased, which correlates with data from the literature for films with a similar composition [27,45]. During storage, GLY can decompose, which can cause the decrease in the breaking hardness of the films, and due to this fact, the water content of the films also decreases. For films with a higher GLY concentration, the decrease in breaking hardness is higher, so these films can become more breakable over time. As we can see, the reduction in breaking hardness was outstanding when comparing the freshly prepared samples and the 3-month samples. As for the 3-month and the 6-month samples, the changes in the breaking hardness values were very low, presumably because most of the chemical decomposition process may take place by the third month. This assumption is also supported by the results of the FT-IR measurements. Because of these facts, it can be said that the films that do not contain CA and contain a low GLY amount have adequate stability in terms of breaking hardness (Samples 1, 5, 9).

#### 3.1.2. In Vitro Mucoadhesivity of Films

**Figure 2:**
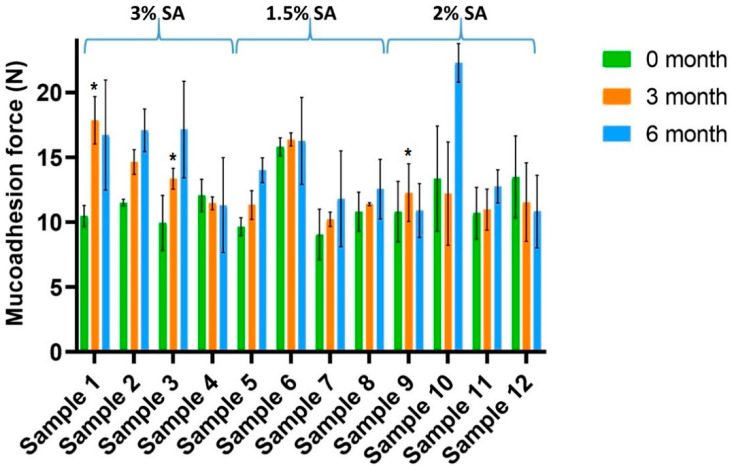
Mucoadhesivity of the prepared films (Samples 2, 4, 6, 8, 10, 12 contain CA) (* *p* < 0.05; *n* = 6). The samples were compared to the freshly prepared sample (0 months).

Figure 2 shows the mucoadhesivity of the films, with different colours at different times. The significance results are marked with * (*: *p* < 0.05). As can be seen, mucoadhesion force increased for almost every sample during the 6-month period. This is a positive fact because it means that the films can connect to the buccal mucosa in a stronger way after storage time. This phenomenon can probably be explained by the fact that more free chains are formed during storage, so carboxyl and hydroxyl groups can connect to the oligosaccharide chain of mucin on the buccal mucosa. This observation is also supported by the results of the breaking hardness of the films because breaking hardness can decrease in every sample during storage, which means the cohesive structure can be destroyed by the forced condition.

Based on our results, SA showed excellent mucoadhesion properties, which correlates with data from the literature [46,47]. Films without HPMC showed a higher rise in mucoadhesion force compared with the films without HPMC (Samples 1–4), so it can be said that HPMC can enhance the stability of the structure formed during the investigated period because the changes of mucoadhesion force in the films containing HPMC are smaller than in the case of the films without HPMC. Moreover, these results suggest that the films containing CA have higher in vitro mucoadhesion force, especially in the case of the 6-month samples, than the films without CA. The GLY concentration can also influence the mucoadhesivity of films. Films with a higher GLY concentration may cause lower growth and growth rate in mucoadhesivity. This observation may be due to the fact that high amounts of GLY can build better in the polymer film structure; therefore, during storage, fewer chains are free that are able to bind to the mucin of the buccal mucosa.

#### 3.1.3. Active Agent Content

**Figure 3:**
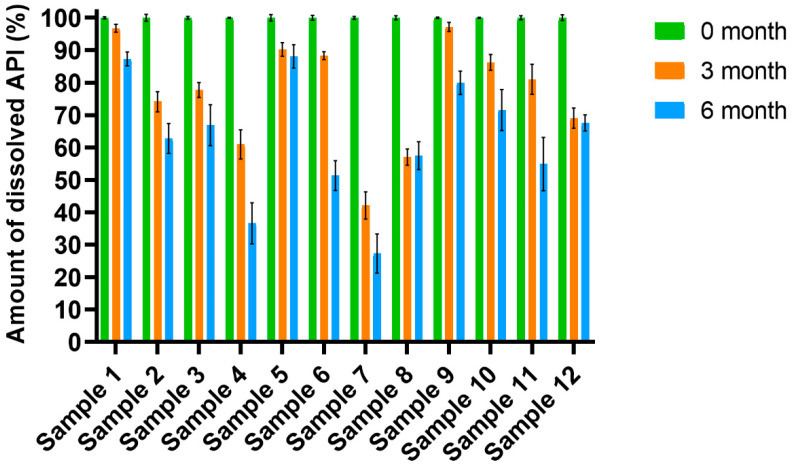
API content for different films during accelerated stability study (Samples 2, 4, 6, 8, 10, 12 contain CA) (*n* = 6).

Figure 3 shows the results of the change in content during 6 months of storage. The results are marked in different colours for the different times of the investigation. In Figure 3, the results appear numerically for the compositions that can preserve more than 85% of the API after 3 or 6 months, according to pharmacopeia expectations. As expected, a decrease can be observed for each sample under the forced condition during storage. The reductions in the amount of the API were higher at the beginning of the investigation, while later, the changes were smaller. The changes were larger for films containing CA, so it can be concluded that CA can decrease the stability of films. GLY can also enhance the decomposition of the API. It was found that films with a larger amount of GLY had a lower amount of API after 6 months than films with a low amount of GLY, so higher GLY concentrations in the films can also reduce film stability. The reason for this is that films with a high GLY concentration have a higher water content because of the water-retention effect of GLY, and a higher water content can increase decomposition. As can be seen, Sample 1 and Sample 5 can preserve the required amount of the API after 6 months, while Sample 6, Sample 9 and Sample 10 can preserve it after 3 months.

#### 3.1.4. FT-IR Spectroscopy

**Figure 4:**
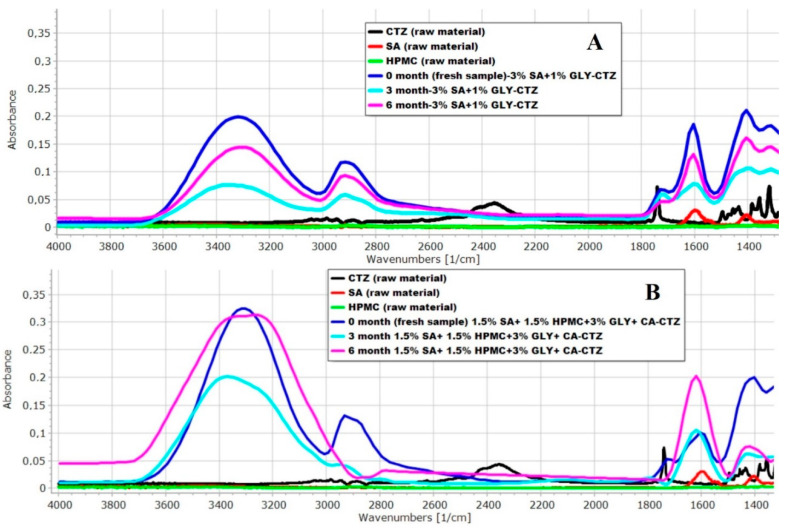
Results of the FT-IR spectra of the prepared films (Part “**A**” Sample 1, Part “**B**” Sample 8).

The prepared films were investigated with FT-IR spectroscopy to determine the interactions between the components of the films. In Figure 4, the FT-IR spectra of the films can be seen. Part “A” of Figure 4 shows the film composition with the largest amount of API (Sample 1) after 6 months, while in the bottom figure, the film composition with the least amount of API is presented (Sample 8). As can be seen, the carboxyl group of CTZ can be detected at 1739 cm^−1^ in the FT-IR spectrum. In the spectrum of CTZ as a raw material, this peak can be sharply distinguished. However, in the films, this peak was shifted to the higher wavenumbers. In Sample 1 (Part “A” of Figure 4), the CTZ peak was shifted towards a higher wavenumber, but the intensity of the peak was smaller than in the case of the raw material. In Part “B” of Figure 4, for Sample 8, the peak disappears completely after 6 months, which shows that the amount of the API decreased because of the forced condition during storage, and hydrogen bonds were formed between SA, HPMC and the carboxylic group of CTZ, which can cause more remarkable structural changes, as seen in the results of the breaking hardness measurement. In addition, there are important bands in the higher wavenumber region. A spectral peak can be found at 2384 cm^−1^, which shows the stretching vibration of the N-H group of CTZ. This peak disappears in every sample, regardless of the GLY concentration. This observation may suggest that interactions develop in the films between the N-H-group of CTZ and the other components of the films. Overall, it can be stated that the decomposition of the API was greater in CA-containing films than in the ones without CA. Moreover, interactions can occur between the components of the films, mostly in the form of hydrogen bonds, which can greatly affect the breaking hardness of the films.

### 3.2. Cytotoxicity Test

The TR-146 cells were treated with the dissolved films in a concentration of 0.35 mg/mL CTZ in HBSS as a solvent. Table 2 shows the results of the cytotoxicity test according to flow cytometry stained with propidium iodide. It can be seen that some formulations had a high impact on cell viability. Samples 7 and 10 were below 40%. However, Samples 3, 5 and 12 had almost no cytotoxic effect. The other samples had a cell viability value between 55 and 87%.

Cellular transport experiments were performed only with Samples 1, 2, 3, 5, 8, 9 and 12, which had cytotoxic values over 60%. 

The direct cytotoxicity of cetirizine dihydrochloride is not well-published. Majumder et al. have found that, in a concentration of 400 µM, mouse macrophage RAW 264.7 and mouse myoblast C2C12 cell lines showed only ~50% and ~60% cell viability, respectively [48]. For human lymphocytes, it was found that a concentration of 200 µg/mL decreased cell viability to 50% [49]. In our cytotoxicity and cell viability experiments, the concentration of cetirizine hydrochloride was higher, 744 µM actually.

### 3.3. Permeation Test

#### 3.3.1. Permeation Test across Artificial Membrane

**Figure 5:**
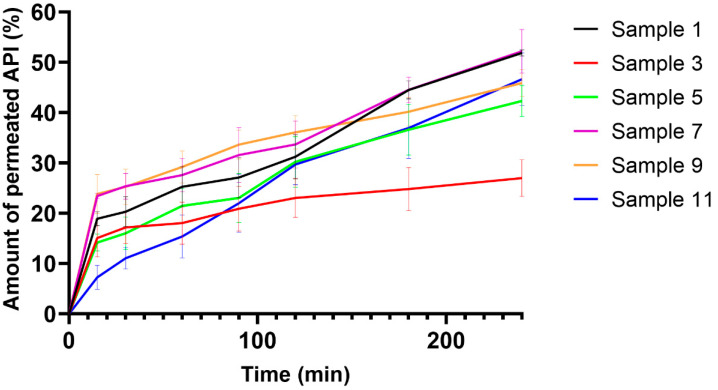
Permeation curves of polymer films on artificial membrane (*n* = 6).

Figure 5 shows the results of the permeation test across an artificial cellulose membrane. The permeation curves of films of different compositions are shown. From most compositions, more than 40% of the API can permeate to the acceptor compartment. The SA concentration can influence the amount of the permeated API. A higher amount of the API can permeate to the acceptor compartment from the films with a higher SA concentration than from the ones with a lower SA concentration. This correlation is shown in Figure 5, where Sample 1 (brown curve) has a higher permeation rate than the comparable samples (Sample 5—purple curve, Sample 9—green curve). The same correlation can be observed for Sample 3 (yellow curve) and Sample 11 (red curve). Furthermore, it can also be stated that the GLY concentration did not influence the permeation rate and speed of the API.

These results can be considered good because the entire amount of the permeated API can have an effect compared to per os tablets because of the first-pass effect of the liver. At the same time, our results can be improved by a greater volume of the donor phase.

#### 3.3.2. In Vitro Permeation Test

**Figure 6:**
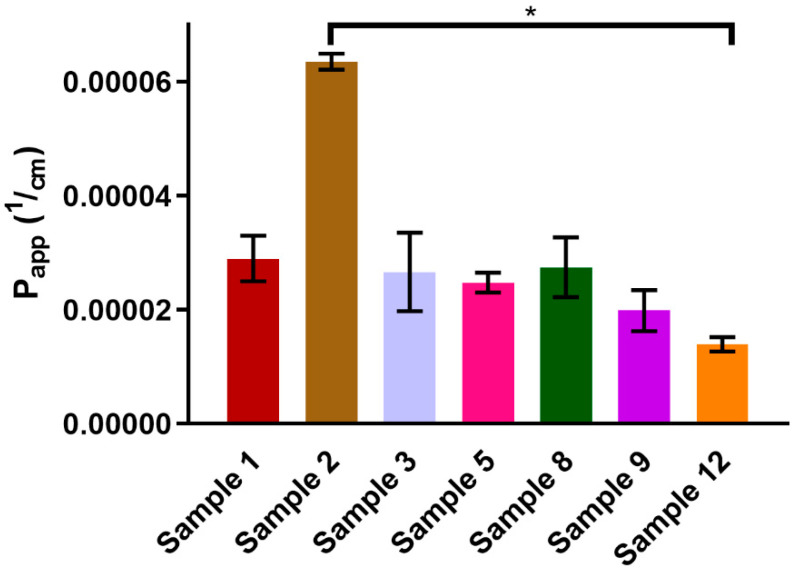
Apparent permeability values of buccal films calculated from the permeability rate of CTZ between 60 and 90 min. Each film was investigated in three separate, parallel cell culture inserts (*n* = 3). * Significant difference was found only between Sample 2 and Sample 12.

TR-146 cells are an accepted model for the in vitro testing of buccal absorption. The prepared films were dissolved in HBSS, and their cellular transports were observed for 90 minutes. As shown in Figure 6, Sample 2 had exceptionally high permeation, while Sample 12 had the lowest value. The presence of citric acid had no direct effect on cellular transport. Moreover, films with HPMC content had smaller permeability.

**Figure 7:**
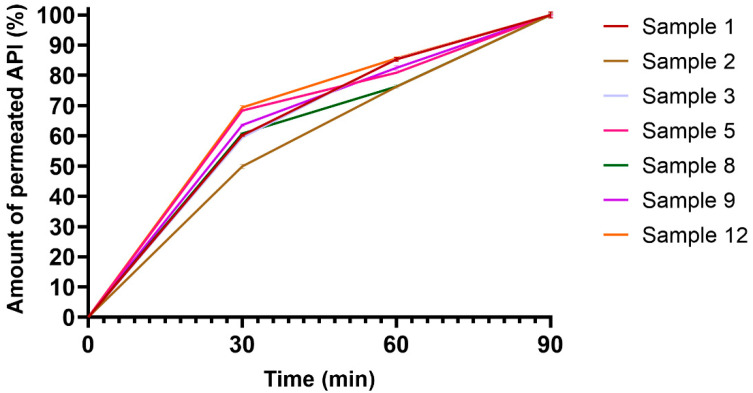
Transported CTZ–time curves of buccal films on the TR-146 cell line. Each film was investigated in three separate, parallel cell culture inserts (Samples 2, 8, 12 contain CA) (*n* = 3).

As can be seen in Figure 7, most films had a linear transport rate through the cells between 30 and 90 min. With the exception of Sample 4, all films had a faster transport rate in the first 30 min, after which the transport slowed. The dissolution and the transport of CTZ from all films was stable, with no observed dose dumping [41].

## 4. Discussion

In our earlier work, we prepared buccal films with CTZ-containing films based on SA and HPMC. We found promising results; therefore, we investigated these films further, and we also formulated new compositions.

In our present research work, we focused on the investigation of the stability and permeability of the previously formulated and CA-containing film compositions. However, new compositions were also formulated and investigated. During the stability test, we investigated the physical (mechanical) and chemical stability of the films. 

The breaking hardness of all films decreased during storage. The decrease was higher for CA-containing films.

However, during storage, the mucoadhesivity of the films can increase to a great extent, which is a positive fact. CA was able to enhance the mucoadhesion force of the films, and the low GLY concentration may also result in beneficial mucoadhesion properties in the films.

Based on the results of the API content, it can be concluded that the CA-containing films have smaller drug content, and the high GLY concentration can also reduce the amount of the API during storage. Nevertheless, there are compositions that can preserve the API content according to pharmacopeia expectations (Samples 1, 5, 6, 9, 10) after 3 or 6 months.

Through the FT-IR investigation, chemical interactions were found in the buccal films between the N-H-group of CTZ and the other components of films. It can be concluded that the decomposition of CTZ is greater in CA-containing films than in the ones without CA. Furthermore, interactions can occur between the components of the films, mostly in the form of hydrogen bonds, which can greatly affect the breaking hardness and mucoadhesivity of films.

Further research is needed to determine which component of the films exhibits cytoprotective properties to decrease the cytotoxic effect of the API. No direct correlation was found between cell viability and the presence of citric acid. The investigation of cytotoxicity in the course of the development of a new pharmaceutical product is essential and unavoidable in order to prove that the product is safe and non-toxic, so it can be used in humans for further tests in clinical trials. In conclusion, it can be said that Samples 3, 5, 8, 9 and 12 have appropriate and acceptable cytocompatibility because they have high cell viability values, so these samples did not significantly impair the cell viability of the films. However, further research is needed to properly evaluate the cytotoxicity of Samples 1, 2, 4, 6 and 11. Samples 7 and 10 proved to be cytotoxic, so further investigation is not justified. 

More than 40% of CTZ was able to permeate to the acceptor compartment from almost all compositions. The SA concentration can influence the amount of the permeated API. A higher amount of the API was able to permeate to the acceptor compartment from the films with a higher SA concentration than from the ones with a lower SA concentration. At the same time, the bioavailability of the API is higher from buccal films than from per os tablets.

The speed of transport of CTZ was nearly constant from all compositions; no excipient caused significantly faster or slower permeation. Apparent permeability was the highest for Sample 2, which was transported across the buccal cell line in a large quantity. CA did not enhance the permeation rate of CTZ in the films, but high alginate content resulted in relatively high permeability. HPMC seemed to decrease apparent permeability. As films with similar compositions were not published before, further investigation is needed to determine how the concentration of each excipient modifies cellular permeability.

Finally, it can be concluded that, from among these compositions, Sample 5 (1.5% SA + 1.5% HPMC + 1% GLY) demonstrated the best stability, cell viability and permeability properties, so this composition is recommended for application on the buccal mucosa. 

## 5. Conclusions

Summarizing our results, it can be said that the breaking hardness of the films decreased during the 6-month period, and the CA reduced it to a greater extent. At the same time, the mucoadhesivity of the films can be increased, especially in case of the CA-containing films. Under storage, the API content decreased, and the CA enhanced the reduction in the API. On the other hand, the high amount of GLY decreased the API content of the films as well. Sample 1 and Sample 5 met the requirements of pharmacopeia after 6 months of stability testing.

Moreover, interactions can be found between the components of the prepared films, mainly in the form of hydrogen bonds.

As a conclusion, it can be said that Samples 3, 5, 8, 9 and 12 have appropriate and acceptable cytocompatibility because these samples have high cell viability values.

Apparent permeability was the highest for Sample 2. CA did not enhance the permeation rate of CTZ in the films, but high alginate concentration resulted in relatively high permeability. 

Finally, it can be concluded that Sample 5 (1.5% SA + 1.5% HPMC + 1% GLY) is recommended for application in buccal drug administration because of its great properties.

## Figures and Tables

**Table 1:** Composition of different SA- and HPMC-based films.

Samples	SA (*w/w* %)	HPMC (*w/w* %)	GLY (*w/w* %)	CA (*w/w %)*	CTZ (10 mg)
1	3	0	1	−	+
2	3	0	1	+	+
3	3	0	3	−	+
4	3	0	3	+	+
5	1.5	1.5	1	−	+
6	1.5	1.5	1	+	+
7	1.5	1.5	3	−	+
8	1.5	1.5	3	+	+
9	2	1	1	−	+
10	2	1	1	+	+
11	2	1	3	−	+
12	2	1	3	+	+

**Table 2:** Cytotoxicity of the prepared films according to flow cytometry. The experiment was carried out in triplicate (Samples 2, 4, 6, 8, 10, 12 contain CA).

Samples	Cell Viability Compared to Control (% ±SD)
1	67.1 ± 2.7
2	78.7 ± 0.6
3	92.8 ± 0.4
4	56.3 ± 1.0
5	99.3 ± 0.8
6	55.9 ± 0.2
7	33.6 ± 0.7
8	87.7 ± 0.1
9	87.2 ± 0.5
10	17.4 ± 0.3
11	56.4 ± 4.6
12	91.3 ± 1.5
Triton X	0.2 ± 0.1

## Data Availability

Not applicable.

## Abbreviations

Sodium alginate, SA; glycerol, GLY; hydroxypropyl methylcellulose, HPMC; citric acid, CA; cetirizine dihydrochloride, CTZ; active pharmaceutical ingredient, API; Fourier-Transform Infrared Spectra, FT-IR; standard deviation, SD; Hank’s balanced salt solution, HBSS; apical compartment, AC; basolateral compartment, BC; apparent permeability, Papp; forward and side scatter, FSC-SSC.
